# Myalgic Encephalomyelitis/Chronic Fatigue Syndrome (ME/CFS): An Overview

**DOI:** 10.3390/jcm10204786

**Published:** 2021-10-19

**Authors:** Undine-Sophie Deumer, Angelica Varesi, Valentina Floris, Gabriele Savioli, Elisa Mantovani, Paulina López-Carrasco, Gian Marco Rosati, Sakshi Prasad, Giovanni Ricevuti

**Affiliations:** 1 Department of Biological Sciences, Faculty of Natural Sciences and Mathematics, University of Cologne, 50674 Cologne, Germany; udeumer@smail.uni-koeln.de; 2 Department of Biology and Biotechnology, University of Pavia, 27100 Pavia, Italy; 3 Almo Collegio Borromeo, 27100 Pavia, Italy; 4 Department of Internal Medicine and Therapeutics, University of Pavia, 27100 Pavia, Italy; valentina.floris01@universitadipavia.it; 5 Emergency Department, IRCCS Policlinico San Matteo, 27100 Pavia, Italy; gabrielesavioli@gmail.com; 6 Department of Neurosciences, Biomedicine and Movement Sciences, Neurology Section, University of Verona, 37129 Verona, Italy; elisa.mantovani@univr.it; 7 División de Neurociencias, Instituto de Fisiología Celular, Universidad Nacional Autónoma de México (UNAM), Mexico City 04510, Mexico; pcarrasco@ifc.unam.mx; 8 Medicine and Surgery, Humanitas University, 20090 Milano, Italy; gianmarcorosati23@gmail.com; 9 National Pirogov Memorial Medical University, 21018 Vinnytsya, Ukraine; sakshiprasad8@gmail.com; 10 School of Pharmacy, Department of Drug Sciences, University of Pavia, 27100 Pavia, Italy

**Keywords:** ME/CFS, immunity, dysbiosis, COVID-19, hormone, depression, genetics, miRNA, therapy, diagnosis

## Abstract

Myalgic encephalomyelitis/chronic fatigue syndrome (ME/CFS) is a chronic systemic disease that manifests via various symptoms such as chronic fatigue, post-exertional malaise, and cognitive impairment described as “brain fog”. These symptoms often prevent patients from keeping up their pre-disease onset lifestyle, as extended periods of physical or mental activity become almost impossible. However, the disease presents heterogeneously with varying severity across patients. Therefore, consensus criteria have been designed to provide a diagnosis based on symptoms. To date, no biomarker-based tests or diagnoses are available, since the molecular changes observed also largely differ from patient to patient. In this review, we discuss the infectious, genetic, and hormonal components that may be involved in CFS pathogenesis, we scrutinize the role of gut microbiota in disease progression, we highlight the potential of non-coding RNA (ncRNA) for the development of diagnostic tools and briefly mention the possibility of SARS-CoV-2 infection causing CFS.

## 1. Introduction

Myalgic encephalomyelitis/chronic fatigue syndrome (ME/CFS) is a complex chronic disease of unknown origin that affects nearly 0.9% of the population worldwide [1,2]. Disease symptoms are often broad, and they overlap with many other conditions, making ME/CFS hard to diagnose. Excessive fatigue, malaise, muscle pain, unrefreshing sleep, dysbiosis, cognitive dysfunction, neuroendocrine and immune alterations are all reported in ME/CFS patients [3]. While ME/CFS is often a chronic condition, some patients can experience periods of partial recovery in between relapses, and disease progression differs largely between patients [4]. Although viral infections have been considered the main trigger of disease onset for a long time, a clear mechanism of pathogenesis is still undefined [5]. It is now becoming clear that ME/CFS origin could instead be explained by a complex relationship between genetic predisposition and environmental factors, with each component contributing to disease manifestation [6]. In this respect, sex, socio-economic status, and age have been reported to correlate with disease presentation, with females being predominantly diagnosed but not necessarily affected more often [7].

Although several consensus criteria have been established in the literature (i.e., Canadian Consensus Criteria, Fukuda, Oxford, International Criteria, etc.) no blood test or diagnostic tool is commercially available [3]. However, the lack of a single set of defined consensus criteria might lead to misdiagnosis. Similarly, a clear therapeutic approach is still lacking. Although different meta-analysis and clinical trials have shown robust evidence in favor of cognitive-behavioral therapy (CBT) and graded exercise therapy (GET) [8,9,10,11,12,13,14,15,16,17], more research should be carried out to find advanced therapeutic approaches [6,18].

Given these limitations, identifying the components which contribute to disease pathogenesis and understanding how they cause disease symptoms may lead to novel diagnostic and therapeutic approaches [6]. Several reviews are available in the literature addressing general and specific topics related to ME/CFS, yet a complete overview of the different aspects leading to disease pathogenesis and progression is still lacking. In this review, we address comprehensively how immune dysfunction, hormonal imbalance, genetics/epigenetics, and cognitive alterations affect ME/CFS patients, providing insights into the emerging role of non-coding RNAs and gut microbiome alterations in disease pathogenesis. Lastly, we also include a brief summary of the potential relationship between the newly coined “long-COVID” and chronic fatigue.

## 2. Methods

To review the role of inflammation, immunity, genetics, epigenetics, cognitive symptoms, dysbiosis, non-coding RNAs, and hormones in ME/CFS, we carried out an exhaustive search in PubMed (U.S. National Library of Medicine) publication database. The following keywords were used alone or in combination: “chronic fatigue syndrome”, “myalgic encephalomyelitis”, “ME/CFS”, “inflammation”, “cognitive symptoms”, “dysbiosis”, “microbiome”, “miRNA”, “non-coding RNA”, “COVID-19”, “long-COVID”, “fatigue”, “therapy”, “diagnosis”, “cytokine”, “genetic”, “polymorphism”, “epigenetic”, “HPA axis”, “depression”, “intestinal permeability” and “infection”. Recent publications were preferred, but no limiting period was imposed in our screening. Furthermore, books, general newspapers, and Institutional Websites were reviewed for possible integration.

## 3. Results

### 3.1. The Role of Inflammation and Immunity in ME/CFS

Like other inter-cellular communication, homeostasis of the immune system is dysregulated in CFS [19]. This means ME/CFS patients will experience symptoms related to immunological changes such as high susceptibility to infections, especially of the upper respiratory tract, long recovery times, chronically swollen and tender lymph nodes, and feeling feverish often [3]. It is not clear yet whether CFS is an inherently low-grade inflammatory disease or whether it is only accompanied by systemic inflammation [5]. The underlying causes for each of the symptoms have not yet been fully elucidated, but the following paragraph aims at summarizing the current state of knowledge in the field.

Multiple changes can be observed concerning the state of inflammation in the body of CFS patients in comparison to healthy people. An inflammatory, cell-mediated immune response is active even when pathogens are absent. This may be an abnormal reaction to common antigens which are harmless [20]. This cell-mediated immune response is generally characterized by a decreased function of natural killer (NK) cells, reduced response of T-cells to antigens [21,22], and persistence of autoreactive cells [23,24,25]. The activated state of the immune system is also indicated by an increase in the biomarker neopterin, which is released by monocytes and macrophages, and a high concentration of acute-phase reactants [5,26]. With impaired NK cell function, the ability of the organism to fight infections decreases. The more severely the function of these cells is impaired, the worse ME/CFS symptoms the patient suffers from typically are, and patients are more likely to contract recurrent infections due to immune suppression [3,27]. One can also observe an expansion of effector memory cells exhibiting type 2 responsiveness which means there is low-grade, chronic inflammation. A phenotypic shift in T-helper cells from Th1- to Th2-cells was already discovered in the early 1990s [3,28,29,30]. T-cells also show increased CD26 surface expression, defective regulatory cell functions, fast exhaustion, and dysregulated cell metabolism [5,19,28,31]. Contradictory studies have been published on whether CFS patients show an increase or decrease in T-regulatory cells [32,33]. Furthermore, CFS patients show persistence of autoreactive cells that can generate autoantibodies during common infections, for example, against ß2-adrenergic receptors and M3 acetylcholine receptors [23,24]. Neutrophils and lymphocytes are more prone to apoptosis than in healthy individuals [34].

The finding of low-grade inflammation is also supported by an altered cytokine profile, pro- as well as anti-inflammatory cytokine levels are reported to be elevated in subsets of patients [5,21]. However, contradictory studies have been published on this topic depending on the methods which were used [21,35]. Cytokine levels which are often reported as increased are IL-1β, IL-1, IL-4, IL-5, IL-6, IL-12 [5], and IL-2 [26,36,37], those which appear decreased are IL-8, IL-13, IL-15, and IL-23 [5,38,39]. Furthermore, TNF-α and IFN-γ levels are increased as well as those of NF-κB, a transcription factor regulated by cytokines such as TNF-α and IL-1β [5,27,37,40,41,42]. As mentioned before, the overall results of these studies are not conclusive, for example for IL-8 and IL-13, increased levels have been reported as well [38]. One of the reasons why these results may differ so much between studies could be the influence of other factors such as sleep, obesity, nutrition, and cognition on the state of inflammation in the body. The time point of measurement during disease progression could also play a role. If changes are observed, they are most pronounced in the first three years of the disease. This is of clinical significance as it enables distinguishing between the early and late stages of ME/CFS [43]. Besides total cytokine levels, the network of cytokine interactions also seems to diverge from the norm [39]. A chronically high level of cytokines may interfere with the stress response to body issues and could partly explain chronic fatigue and flu-like symptoms in many patients’ experiences.

It is not clear what exactly causes the onset of symptoms in ME/CFS, but viral infections and stress have been discussed as a possible origin of the disease, while an additional genetic component is also likely [3,44,45,46]. Infectious pathogens such as viruses could be the original cause of the inflammatory state by activating antiviral immune responses, which then trigger systemic inflammation [5,26,34,45]. The virus infection most widely reported in relation to CFS is Epstein-Barr virus (EBV) since a considerable number of patients report symptom onset after contracting EBV [47,48,49]. However, it should be noted that an estimate of >90% of the adult population generally test positive for past EBV infection, and most do not develop ME/CFS. Human herpesvirus 6 (HHV-6) and human parvovirus B19 have also been reported as possible causes of CFS [50,51,52,53,54]. The probability of patients developing CFS after severe viral infections or other illnesses such as Lyme disease has consistently been reported as 5% to 10% [55]. Regardless of which virus infection may trigger ME/CFS, specific immunological changes that CFS and viral infections have in common include altered antiviral response elements, for example, the 2-5A synthetase/ribonuclease L (RNase L) antiviral defense pathway in monocytes, which is mediated by interleukins [3,31,56], and elevated cytokine levels. RNase L then destroys cell membranes in CFS patients, including mitochondrial membranes which causes additional oxidative stress [57]. When suffering acutely from a sore throat, patients very often present with a viral reactivation, which may also be accompanied by tender, swollen lymph nodes [3]. Besides viral infections, another possible explanation for dysregulation of the inflammatory cascade is impairment of the hypothalamus–pituitary–adrenal (HPA) axis, since the systemic hypocortisolism which has been reported in this respect is known to impact immunological homeostasis and drive Th2-cell identity [28,58] (see also HPA axis paragraph). Patients who received cognitive behavioral therapy (CBT) showed lower cortisol levels after treatment compared to untreated patients [58].

Multiple studies have reported elevated levels of oxidative stress in CFS patients [5,34,59]. The antioxidant capacity seems to be decreased in subgroups of patients, but even in patients with normal antioxidant capacity, an increase in oxidative stress is observed. The activity of oxidative and nitrosative pathways is enhanced while levels of antioxidants such as zinc and enzymes like coenzyme Q10 are decreased [5,21,60,61]. This may lead to excessive formation of free radicals, which cannot be eliminated and will damage the cells by targeting fatty acids and proteins [5,21]. These are then recognized as abnormal by the immune system and may in part lead to a chronic inflammatory state. An IgM-mediated immune response directed against O&NS-modified epitopes in ME/CFS has been observed [5,61]. In this context, mitochondrial dysfunction, which has been observed as well [5], can also play a role as this organelle is crucial for reactive oxygen species (ROS) regulation. ROS-induced damage to the mitochondria and elevated pro-inflammatory cytokines, which are both also consequences of viral infection, can activate NF-kB transcription.

Since the inflammatory signaling pathways generally seem to be disturbed, one possible explanation for the symptoms of the disease could be a disrupted gut barrier [45]. The leaky gut hypothesis is supported by the finding that IgA levels in CFS patients against lipopolysaccharides (LPS) of gram-negative bacteria are increased, which is accompanied by increased translocation of these bacteria [5] and the fact that CFS and irritable bowel syndrome (IBS) often occur together [62]. Another possible explanation for the pronounced immune response could be autoimmunity. A few factors support this idea, such as a high prevalence in women which is common in autoimmune diseases, the increase in baseline inflammation, and that it often occurs as a comorbidity of other autoimmune diseases [3,47,55,63,64]. As mentioned above, CFS can occur after infection with EBV, which is also a known risk factor for developing autoimmune diseases [65,66]. If patients are treated against autoantibodies, the condition improves [25,64]. What strongly speaks against the idea of CFS being an autoimmune disease, however, is the lack of tissue damage.

Besides these differences in the baseline state of the immune system in CFS patients compared to healthy people, patients also experience post-exertional malaise (PEM) [55,67,68]. One possible explanation for this might be a more pronounced immune response in ME/CFS patients after exercise than in healthy people [69]. Upon exercise, physically but also mentally, symptoms usually worsen within 24 h, however, there is contradictory evidence against an immune response that diverges from the norm, possibly due to differences in study design [68]. Reports have been made of an increase in TLR-4 and IL-10 gene expression after exercise [69]. While the gene expression was increased, the circulating cytokine levels in response to exercise appear to be similar in CFS and control groups in some studies but diverge strongly in others [69,70]. When studying the complement response to exercise in CFS patients, some evidence was found for a stronger response than in controls. This is of interest because an altered complement response might cause PEM [69]. Moreover, patients seem to suffer from increased oxidative stress faster and longer after exercise than healthy controls, and their antioxidant response is delayed and reduced [46,59]. This fits with the higher level of oxidative stress in these patients even without exercise and supports the hypothesis that some of the symptoms are caused by malfunctions in ROS regulation. Further findings point towards decreased ATP levels, increased lactate, hyperactive RNase L activated by IFN, and hyperactive NF-κB relative to healthy controls. Overall, the evidence suggests that the immune response of CFS patients to exercise is more pronounced than that of healthy people [70].

### 3.2. Genetic and Epigenetic Alterations

Although CFS pathogenesis is still largely unknown, several studies suggest the possibility of a genetic predisposition. First hints came from the observation that mothers and children diagnosed with CFS share very similar symptoms, in contrast to fathers and their children [71]. Moreover, the analysis of data obtained from the Utah health care system highlighted a strong contribution in favor of CFS heritability [72]. Many pathways have then been linked to disease symptoms and severity, such as regulatory pathways of immunity and neurotransmission, inflammation and oxidative stress, the catecholamine pathway, and the serotoninergic system [73] (Table 1). TNF-α, IL-1β, IL-4, IL-6, HLA, IFN-γ, GRIK2, SCL6A4, COMT, and NR3C1 genes have all been found to be correlated with the disease [73]. For a summary of the most significant findings regarding CFS and genetic predisposition please refer to Table 1.

Despite most studies reporting the association between CFS and one or a few polymorphisms, it should be noted that, being a multifactorial disease, a varied genetic contribution is more likely to explain predisposition and heredity than a single variation. In this respect, many variations scarcely contribute by themselves, but when put together they increase the risk. Thus, searching for haplotypes or combined genetic polymorphisms will be helpful in establishing a genetic screening test able to diagnose and/or stratify CFS patients [74]. Possibly, this could also be useful for the administration of personalized and tailored therapy [75].

Genetic predisposition has also been hypothesized to be involved in autoimmunity. Blomberg et al. present a model in which, following infection, certain genetic backgrounds and dysbiosis might favor the generation of B-cell clones prone to react against self-antigens, thus explaining why some patients present signs of autoimmunity [46].

Besides classical genetics, a growing body of evidence suggests that epigenetics is also linked to CFS and can potentially explain the major pathways involved in the disease. In one study, methylation patterns of 10 CFS patients have been compared to 10 controls, and immune, metabolic and neurological pathways have been associated with the disease [4]. Moreover, differential methylation in the PRF1 gene and in several CpG loci of T lymphocytes was also detected in CFS patients in contrast to healthy subjects [76,77]. Perhaps not surprisingly, the genetic and epigenetic alterations found in CFS often reside in the same genes and affect the same functions, thus confirming the importance of the previously mentioned pathways in disease pathophysiology.

Although the new discoveries in CFS and genetics are rising in expectancy in terms of new diagnostic and therapeutic tools, it should be considered that studies with a higher number of participants are needed to achieve true significance. Indeed, independent research is usually conducted on a very limited number of CFS cases and analysis on different patient cohorts often fails to reproduce matching results [74]. Therefore, while the recent data can certainly increase our knowledge of disease mechanisms and has translational potential, more confirming evidence is needed before applying this knowledge in clinical practice.

### 3.3. Cognitive Symptoms and Depression

It is well known that cognitive symptoms such as sleep disorders, depression, anxiety, and mood swings are often found in and characterize CFS. Indeed, a recent systematic review and meta-analysis reported that around half of the ME/CFS patients present with anxiety and/or depression [89].

Diagnosis of CFS is achieved by using well-established diagnostic criteria (Canadian Consensus Criteria, Fukuda, Oxford, International Criteria, etc.). In this respect, carefully defining the forms of associated chronic fatigue (i.e., in cancer, multiple sclerosis, inflammatory bowel disease, psychiatric conditions) is critical to reaching a conclusive diagnosis. Typically, a detailed medical history of the patient including symptoms, the associated disability, the choice of coping strategies, and the patient’s own understanding of their illness are considered. Since CFS and major depression (MD) share very similar characteristics, many CFS patients are initially diagnosed as depressed [80]. Although the diagnosis of MD should be an exclusion criterion for ME/CFS, distinguishing between MD and reactive depression, which can be a comorbidity of CFS, is not always easy. However, while the two conditions show some similar symptoms, they can still be distinguished. For example, in depressed people, fatigue is associated with apathy, whereas in CFS patients it is associated with intense frustration about their condition [90]. In addition, every CFS evaluation should include a mental status examination to identify abnormalities in mood, intellectual function, memory, and personality changes. Particular attention should be directed toward acute depressive, anxious or self-destructive thoughts and observable signs such as psychomotor problems. Moreover, a physical examination may show a frequently sore throat and tender cervical or axillary lymph nodes in CFS, which are not found in depression [90].

As briefly mentioned, not only is there a clear symptom overlap, but several articles also show that ME/CFS and MD can be defined as comorbid [90]. Multiple reasons for this co-occurrence can be discussed. For example, one of the main symptoms of CFS/ME is chronic pain of differing quality and fatigue, and depression is a comorbidity of pain itself [91]. Another possible reason for this comorbidity may be immune system dysregulation, as discussed above. Patients with ME/CFS have poorly functioning NK cells, which is linked to the severity of the illness and disturbed cognitive function, while low NK cytotoxicity has also been found in other diseases including MD disorder [3]. Recent works have also found that, during chronic inflammation, microglia are activated and participate in creating a neuro-inflammatory environment that is similarly found in patients with depression [92]. Leaky gut and metabolic endotoxemia may also explain MD and CFS symptom overlap. Recent studies demonstrated that both diseases show activated immune-inflammatory pathways, including increased Gram-negative bacteria translocation and higher levels of pro-inflammatory cytokines, such as IL-1 [93]. Interestingly, in chronic depression increased levels of IL-1 are associated with higher levels of fatigue and psychosomatic symptoms, including hyperalgesia, insomnia, and neurocognitive deficits [94]. Furthermore, depression sometimes also results from CFS. Poor concentration, groping for words, short-term memory loss, and reading impairment are reported in CFS patients, with severely affected patients experiencing strongly disabling cognitive symptoms [3,95]. This complex psychological condition often prevents patients from continuing their normal lives, leading to severe depression that in turn may worsen the already serious cognitive symptoms.

However, not all CFS patients present with depression. Clinical reports of CFS patients without a history of depression show that antidepressant treatments may even be harmful in these cases [3,90]. Although clinical diagnosis based on symptom manifestation is certainly fundamental, results of some studies suggest that diagnostic tools based on molecular and biological analysis could improve the diagnosis. While further investigation is needed, Table 2 summarizes the proposed biomarkers based on which diagnostic tools could be created to distinguish between CFS and MD (Table 2).

It should be noted that the neuropsychological conditions described in ME/CFS have been hypothesized to originate at least in part from neuroinflammation. Higher levels of proinflammatory cytokines have been found in the cerebral spinal fluid of CFS patients than in healthy controls, and activation of microglia and astrocytes has been verified by positron emission tomography (PET) scan. Over-activity of microglia and astrocytes showed a correlation with symptom severity in patients. However, MD cannot be attributed to neuroinflammation [96,97,98].

To date, there is no standard therapy available that will effectively alleviate symptoms of the disease. There are, however, different approaches that have been tried in the past which appear promising. Classical approaches are exercise treatment to slowly build increased resistance to fatigue, and cognitive behavioral therapy (CBT) to alleviate the psychological strain of the disease [3,55,58]. A major concern in CFS is chronic pain treatment.

In this respect, meditation and relaxation response, warm baths, massages, stretching, acupuncture, hydrotherapy, chiropractic, yoga, Tai Chi, TENS (transcutaneous electrical nerve stimulation), physiotherapy and nerve blocks have all been proposed, but their efficacy is still unclear [3]. Although mild pain killers and non-steroidal anti-inflammatory drugs (NSAIDs), such as ibuprofen and naproxen, can be used in clinical practice to temporally relieve headache, muscle pain, and fever, they often fail to treat chronic pain, thus not providing relief in the long term [107,108,109]. Moreover, evidence for their efficacy as adjuvant medicine in ME/CFS treatment is still lacking [110], and no large-scale clinical trials support their prescription. A possible explanation for the lack of efficacy in ME/CFS should be sought in the origin of pain in these patients [111]. In this respect, central sensitization, which is pain hypersensitivity due to amplification of neuronal signaling, may play a major role. The nervous system is tuned to high pain reactivity, resulting in hyperalgesia. Neurological changes can be observed in these patients such as the ectopic firing of dorsal root ganglia cells or anatomical changes to neurons and the dorsal horn. Neuroinflammation can possibly contribute to central sensitization. NSAIDs may still be used to modulate the activity of nociceptors, but antidepressants have shown higher efficacy in managing this type of pain while physical therapy and psychotherapy are also helpful [112,113].

Oftentimes, doctors advise CFS patients to rest physically. However, it is important to point out that patients, especially those with a depressive disorder and no contraindications for physical stress, should be recommended to undergo structured and supervised physical training, as exercise therapy has been shown to improve symptoms in some patients [114]. Data from eight randomized clinical trials concluded that physical therapy improves exhaustion, quality of sleep, and health status of the patients in the long term, thus showing beneficial potential [12]. This finding contradicts the widespread opinion that patients always feel uncomfortable after physical exertion, a phenomenon known as PEM [115]. One of the aims of CFS treatment is the prevention of depression and suicidal tendencies by managing the physical and emotional issues resulting from ME/CFS [3]. Short-term studies of CBT in CFS have shown improvement in function and symptom management, especially in conjunction with other treatment modalities and compared to relaxation controls [116]. Symptoms of fatigue decreased mood, and physical fitness have been shown to be significantly ameliorated in patients following CBT [9,11,14,15], even in children and adolescents [10,117,118]. Moreover, the ability of CBT to relieve pain in ME/CFS has also been reported [119]. However, the outcome of CBT for CFS as a psychotherapeutic intervention and CBT effectiveness in improving cognitive function and quality of life still need to be fully addressed, as some gaps remain in the current evidence base [120,121,122,123,124].

Considering the overlap with depression which has already been discussed, it is not surprising that antidepressants might also be useful in treating mental aspects of CFS [124]. A large meta-analysis including 94 studies showed that antidepressants were approximately 3.5 times more effective than placebo in treating chronic pain in CFS patients [125]. Fluoxetine, for example, has shown the ability to improve symptoms and immune function [126] while Bupropion was found effective for the treatment of fatigue and depression in nine fluoxetine resistant CFS patients [127]. However, some authors underline the lack of studies on the efficacy of antidepressants in treating ME/CFS, and more studies on different antidepressant molecules should be carried out before establishing a therapy [128].

### 3.4. HPA Axis and Hormonal Imbalance

When suffering from ME/CFS, patients can experience dysregulation in the levels of hormones produced by the HPA axis [129]. Indeed, despite the heterogeneity of symptoms affecting CFS patients, and the evidence of multifactorial pathogenesis, a hormonal imbalance has been demonstrated to have a direct link with some of the symptoms present in CFS, such as debilitating fatigue, difficulty with concentration, and disturbed sleep [130]. In this respect, meta-analysis evidence supports the presence of hypocortisolism in CFS patients. Cortisol levels are fundamental to maintaining hormone homeostasis, and when altered they may cause metabolic, inflammatory, and memory alterations, although it is not sure if inflammation is a cause or consequence of a hormonal imbalance. Moreover, a loss of morning peak ACTH (adrenocorticotropic hormone) and decreased responsiveness to pharmacological challenge are also reported in CFS cases compared to controls [130]. Several symptoms of CFS resemble those of hypothyroidism caused by lower thyroid hormone activity that may be due to underlying chronic inflammation. A case-control study demonstrated that chronic fatigue syndrome patients exhibited lower FreeT3 (triiodothyronine), TT3 (total triiodothyronine), decreased peripheral conversion of T4 (thyroxine) to T3, normal/high-normal TT4 (total thyroxine) level, and lower protein binding of thyroid hormones [131].

It is well-known that the prevalence of CFS is substantially higher in females compared to men. Moreover, women with CFS have a significantly greater probability of reporting an earlier onset of menopause due to any gynecological surgeries (hysterectomy and oophorectomy) as well as pelvic pain and associated endometriosis compared to controls. The consequences of a hysterectomy and early onset menopause will bring about a decline in sex hormone levels. Low levels of estrogen can affect the immune system, causing chronic fatigue and sleep disorders. Indeed, as the delicate balance between estrogen and progesterone is lost, an improper inflammation response can arise [132].

It is now clear that proper function of the HPA axis is important for homeostasis. As patients present with changes in the HPA axis, it is reasonable to wonder about possible neuroendocrine implications in CFS etiopathogenesis. However, the main question is whether HPA alterations are implicated in the genesis of the disease or if they are secondary to the development of CFS. In this respect, it would be worth investigating which role is played by hormonal imbalance in disease pathogenesis [133]. One popular hypothesis is the so-called “allostatic load condition”, where the neuroendocrine system responds to a stressor (allostatic state) in order to reset the physiological set-point (homeostasis). If this mechanism fails, an allostatic overload takes place, and the way the body deals with the stressor perpetuates stress and the chronicity of the condition [134]. Possibly, this situation may proceed dysfunction of the HPA axis. However, clear evidence in support of this suggestion is still lacking, and more studies need to be carried out to understand the role of neuroendocrinology in CFS pathogenesis [135].

### 3.5. Dysbiosis and Intestinal Permeability

Several papers have pointed out an alteration in the gut microbiome composition in CFS patients, and involvement of dysbiosis in disease pathogenesis has been hypothesized [136,137,138]. In particular, a decrease in microbial diversity and a drop in Firmicutes number was found in CFS patients compared to controls [137]. Moreover, other studies confirmed a reduction in Bacteroidetes/Firmicutes ratio and an increase in Enterobacteriaceae, thus providing evidence of a complete reorganization in intestinal microbiome composition and function [139,140]. The use of microbiota alteration as a diagnostic biomarker has also been suggested, but disease overlap with other intestinal disorders may represent a disturbing factor during diagnosis and patient stratification [138]. Although the gut microbiome is crucial in different disorders, the role of dysbiosis in CFS pathogenesis remains to be fully addressed, and its role in this disease is still up to debate [141]. After 18S RNA sequencing in the stool of 49 ME/CFS patients and 39 healthy individuals, Mandrano et al. reported a nonsignificant difference in eukaryotic diversity [142]. Therefore, more studies are needed to fully understand gut microbiome involvement in disease pathogenesis and progression.

Dysbiosis is a well-known cause of increased gut permeability. This phenomenon, also known as leaky gut, allows bacterial translocation into the bloodstream, thus increasing systemic inflammation via an immune response mediated by higher levels of LPS derived from Enterobacteriaceae [26,143,144,145]. More commensal bacterial translocation and increased gut inflammation have been reported in ME/CFS cases when compared to healthy controls, similar to what has already been found in obesity, diabetes, metabolic syndrome, nonalcoholic fatty liver disease, and septic shock. [143,145,146,147,148]. Therapeutic interventions aimed at re-establishing eubiosis and reducing intestinal permeability may be helpful in this respect. It has been demonstrated that a leaky gut diet, together with anti-inflammatory and anti-oxidative substances, is able to significantly improve CFS conditions [149]. Moreover, the use of probiotics and/or prebiotics should also be considered, and preliminary studies in mice and rats show promising results [150,151,152,153]. Finally, positive outcomes were reported using fecal microbiota transplantation (FMT) in CFS patients [154], but further evidence is needed. In addition, several concerns about FMT, for example, a lack of consistency, donor problems, long-term safety, etc., still raise doubts about safety and feasibility, limiting its use in the clinical practice [155,156,157,158,159].

Altogether, these data point out that intestinal microbiome involvement in disease pathogenesis and progression should be further analyzed, and that promising novel therapeutic tools targeting leaky gut and dysbiosis could potentially arise for CFS patients.

### 3.6. Non-Coding RNAs

Non-coding RNAs (ncRNA) control various levels of gene expression, chromatin architecture, epigenetic memory, transcription, RNA splicing, editing, and translation [160]. One specific type of ncRNA, the microRNAs (miRNA), alter and modulate several developmental, physiological, and pathophysiological processes [161]. This modulation can be achieved in different ways: by silencing genes, by initiating the cleavage of their respective target mRNA, or by inhibiting gene translation after complete or partial binding to their target sequence [162].

Altered protein expression characterizes chronic pain and contributes to the development of long-term hyper-excitability of nociceptive neurons in the periphery. Moreover, the central nervous system is characterized by expressional changes of signaling molecules, transmitters, ion channels, or structural proteins [163]. As miRNAs are part of mechanisms of gene expression, they are likely to contribute to these changes.

There is a need for unbiased, specific diagnostic biomarkers for ME/CFS to expedite patient diagnosis and treatment, as some previously proposed biomarkers such as activin B are controversially discussed [164,165]. miRNA profiles represent a promising strategy to discover biomarkers and more recently to diagnose patients. A limitation for the biomarker discovery studies in ME/CFS is the low number of participants that have been recruited. Patients with ME/CFS show differential expression of miRNA coding genes that regulate cytotoxicity, cytokine secretion, and apoptosis [166]. Therefore, miRNAs have the potential to be utilized as biomarkers for disease diagnosis and prognosis, but it is imperative to find a way to make markers as accurate as possible for the patient, considering their gender, age, and lifestyle. Indeed, it has previously been shown that differential expression of miRNAs in ME/CFS depends also on gender, exercise, and disease state. It is extremely important to align assessment and reporting with Common Data Elements (CDE) in human subject research to improve data quality that allows for comparisons across multiple studies [167]. The pathways in which each miRNA exerts its activity are not clear yet, but several miRNAs have been identified as altered in ME/CFS patients.

Most of the miRNAs differentially expressed in patients with CFS are involved in immune response regulation. For example, up-regulation of miR-150-5p is seen in both T-cell and B-cell maturation and differentiation and influences the release of pro-inflammatory cytokines. MiR-199-3p is a negative regulator of NF-κB and IL-8. Low miR-199-3p expression, seen in ME/CFS subjects, is linked with poor survival outcomes in carcinomas, possibly affecting the disease-related physiological burden. Another dysregulated miR-223 modulates the TLR4/TLR2/NF-κB/STAT3 signaling pathway consequently affecting inflammatory cytokine expression [161]. The cytokines released in response to the inflammatory assault, particularly TNF-α, are directly suppressed by miR-130a-3p, reducing inflammation, and associated oxidative stress. MiR-146a regulates the expression of STAT1 and reduces IFN-γ secretion, resulting in the loss of the repressive effect of regulatory T lymphocytes, while miR-374a-5p regulates the expression of ubiquitin ligase, mTOR signaling pathway, and monocyte chemoattractant protein (MCP)-1, critical in inflammatory and immune response. The overexpressed miR-4443 increases pro-inflammatory cytokines by activated the NF-κΒ pathway via targeting TRAF4. The expression of miR-558, miR-146a, miR-150, miR-124, and miR-143 associates directly with higher expression of immune inflammatory-related genes encoding TNF-α, IL-6, and COX-2 in adolescents with CFS [161]. In addition, NK cells have demonstrated the greatest changes in miRNA expression with upregulation of hsa-miR-99b and hsa-miR- 330-3p. This is consistent with the ME/CFS phenotype characterized by NK cells activity alterations [168].

Another important factor in ME/CFS is endothelial function. Silent information regulator 1 (Sirt1) reduces inflammation and oxidative stress and increases the production of nitric oxide by activating the endothelial nitric oxide synthase. MiR-21, miR-34a, miR-92a, miR-126, and miR-200c regulate endothelial function via the Sirt1/eNOS axis but it is necessary to further explore how this regulation occurs and its effectors [169].

In 2020, a new technique consisting of a post-exertional stress challenge that provokes PEM in ME/CFS patients was developed, allowing to obtain measurements of the differential expression of circulating miRNAs in severely affected patients. This study led to the discovery and validation of eleven miRNAs associated with ME/CFS and the creation of a machine learning algorithm that allows the classification of ME/CFS patients into four clusters associated with symptom severity, providing a foundation for the development of a new non-invasive test to diagnose ME/CFS. These miRNA signatures and clusters could potentially be used to predict responses to pharmacological treatments for ME/CFS and may even allow clinicians to identify individuals for whom such treatments could be beneficial [170].

MiRNAs are not the only type of ncRNA with a promising role in CFS diagnosis and prognosis. Emerging roles of long non-coding RNAs (lncRNAs) in immune regulation and disease processes are being discovered. The levels in peripheral blood mononuclear cells (PMBCs) of NTT and EMX2OS (two lncRNA associated with immune response) have been associated with more severe ME/CFS, suggesting a potential diagnostic value of these lncRNAs. For NTT, it has been proposed to exert its function on nearby genes involved in cell proliferation, apoptosis, or inflammation, due to its large size (17 kb). A marked positive correlation between NTT and IFNGR1, another lncRNA, was observed in ME/CFS, suggesting that the NTT/IFNGR1 axis might play role in disease pathogenesis. The expression level of EMX2OS was found to have elevated PBMCs from CFS patients. The role of EMX2OS in PBMC is currently unclear and requires more experiments to be elucidated [171].

Together, the previously mentioned studies provide a basis to develop an integral diagnosis and prognosis program which not only includes metabolic analytes but also molecular ones, such as miRNA or lncRNA, for diagnosing and choosing the best treatment for ME/CFS patients.

### 3.7. ME/CFS and COVID-19

As of August 2021, the Coronavirus disease 2019 (COVID-19) outbreak caused by the Severe Acute Respiratory Syndrome Coronavirus 2 (SARS-CoV-2) has led to nearly 216 million cumulative cases, with 4.5 million deaths worldwide (WHO, 2021, https://www.who.int/publications/m/item/weekly-epidemiological-update-on-covid-19---31-august-2021, accessed on 31 August 2021).

The clinical interest recently shifted from the acute to the chronic COVID-19 phase which is causing additional disease management issues. Indeed, a proportion of COVID-19 survivors fail to revert to their preexisting condition and report persistent debilitating symptoms likened to CFS several months after COVID-19 acute infection resolution [172,173,174]. This chronic post-viral syndrome has been termed as “long-COVID” or “post-acute COVID-19 syndrome” and has been reported to affect patients irrespective of the severity of the acute infection [175]. However, it should be noted that the term “long-COVID”, although widely used now, is still poorly defined, as multiple entities beyond chronic fatigue are included, thus raising questions about the conclusiveness of studies on long-COVID. In this respect, basic research on the underlying molecular and cellular mechanisms can be of great help in defining more about post-COVID-19 and ME/CFS symptoms relationship. Estimates of long-COVID vary widely based on the timing of follow-up. One study reports that nearly 90% of 143 patients experienced at least one symptom, in particular fatigue and dyspnea, two months after acute infection recovery [176]. The percentage of patients with persistent symptoms at nine months follow-up was reported to have dropped to 30%, according to a longitudinal prospective cohort study also including outpatients with mild acute disease course, with fatigue, loss of smell and taste and, “brain fog” being among the most common referred complaints [177].

With a wide array of symptoms centered around fatigue, brain fog, diffuse myalgia, non-restorative sleep, and depressive symptoms, long-COVID resembles ME/CFS, which is frequently associated with viral infections [178,179]. Interestingly, clusters of ME/CFS-like symptoms have been observed following other coronavirus outbreaks, including SARS in 2001 and MERS in 2012 [180]. Reduced quality of life and persistent pain and fatigue were reported at 6 months after hospital discharge in 30% of SARS and MERS survivors [181]. Besides this, one study reported that 27% of SARS survivors met the criteria for ME/CFS 41 months after infection [182]. Moreover, in a recent meta-analysis of post-infectious symptoms following SARS and MERS, fatigue was the most debilitating symptom in 19.3% of patients up to 39 months after infection resolution [183].

The prevalence and duration of long-COVID symptoms resembling ME/CFS are still under investigation and there are some uncertainties because of heterogeneous patient populations, follow-up duration, and inclusion criteria [184]. Only a few studies so far have applied ME/CFS diagnostic criteria. A retrospective analysis reported that 85.3% of 231 COVID-19 survivors gathered from the Genome Database of Latvian Population national biobank reached the threshold for ME/CFS diagnosis, with three or more long-term ME/CFS-like symptoms persisting at 6 months follow-up [185]. A single-center prospective longitudinal study found that only 13% of 130 patients with moderate-to-severe COVID-19 pneumonia met the criteria for ME/CFS 6 months after discharge [186]. In a small, single-center pilot study, ME/CFS-like features were found in 27% of 37 COVID-19 survivors, six months after recovery, with no difference in clinical inflammation, lung function, serum neurofilament light chain (a biomarker of axonal damage), and objective cognitive testing when comparing patients with versus without ME/CFS-like features [187]. Another study found that 14.2% of 120 COVID-19 survivors met the ME/CFS diagnostic criteria 6 months after infection onset [188]. A case series described ME/CFS-like patterns after COVID-19 infection resolution in three adolescents and young adults 6 months after recovery [189].

Despite the similarities between the symptoms of long-COVID patients and ME/CFS, further evidence is required to list COVID-19 among the infections associated with ME/CFS. Last, additional investigations with longer follow-ups, more uniform criteria for ME/CFS diagnosis, including both in- and outpatients with infections of different severity and a control group of people affected by other infections are required to better characterize risk factors, prevalence, and progression of long-COVID ME/CFS-like features, and to design specific interventions and treatments.

## 4. Discussion

Altogether, the insights presented show that ME/CFS is a complex systemic disease that affects many organs. By reviewing the most important pathways and systems associated with disease pathogenesis and symptoms, our review encourages to account for ME/CFS as a multifactorial disease that cannot be diagnosed or treated appropriately if it is not considered in its entirety. Consequently, any diagnostic method based on blood tests or biomarkers needs to take into account disease heterogeneity and complexity. Moreover, inter-individual variability in ME/CFS manifestations is striking and should be considered when developing novel therapeutic tools. Personalized and tailored approaches should be preferred to a one-size-fits-all therapy in this respect, but much remains to be elucidated to define specific patient subgroups.

Although more studies are urgently needed, our summary provides a general overview that can be useful to provide a better understanding of ME/CFS pathogenesis and to find new diagnostic/therapeutic opportunities for a disease that, although strongly debilitating, is still largely unexplored.

## Figures and Tables

**Table 1:** Summary of the most significant genetic alterations found in CFS patients.

Ref.	N° Patients	Gene/Protein	Alteration	Pathway
Smith et al., 2011 [78]	40 CFS + 40 controls	GRIK2 (glutamate receptor, ionotropic, kinase 2)	G allele of rs 2247215 (GRIK2)	Glutamatergic neurotransmission
		NPAS2 (neural PAS domain protein 2)	T allele of rs 356653 (NPAS2)	Circadian rhythm regulation
Schlauch et al., 2016 [79]	42 CFS + 38 controls	CLEC4M (C-Type lectin domain family 4 member M)	C > T missense mutation (CLEC4M)	Signal transduction and kinase reaction
		GRIK3 (glutamate ionotropic receptor kainate type subunit 3)	CT genotype at rs3913434 (GRIK3)	Glutamatergic neurotransmission
Meyer et al., 2015 [80]	120 CFS (12–18 years) + 38 controls	SCL6A4 (solute carrier family 6 member 4), encodes for 5-HTT	SNP rs25531 A > G and short (S) vs. long (L) 5-HTTLPR allele	Serotonin reuptake
Lobel et al., 2015 [81]	74 CFS + 76 controls	COMT (catechol-O-methyltransferase)	rs 4680 polymorphism	Catecholamine inactivation
De Luca et al., 2015 [82]	89 FM/CFS + 196 controls	NOS2A (nitric oxide synthase 2A)	NOS2A −2.5 kb (CCTTT)_11_ allele	Inflammation and oxidative stress
Fukuda et al., 2013 [83]	155 CFS	GCH (GTP cyclohydrolase I)	C+243T polymorphism (GCH)	tetrahydrobiopterin (BH4) biosynthesis
		TH (tyrosine hydroxylase)	C-824T polymorphism (TH)	Catecholamines biosynthesis
Smith et al., 2008 [84]	40 CFS + 55 with insufficient fatigue + 42 controls	HTR2A (5-hydroxytryptamine receptor 2A)	-1438G/A, C102T and rs1923884	Serotoninergic system
Carlo-Stella et al., 2006 [85]	54 CFS	TNF promoter	-857 TT and CT genotypes (TNF)	Inflammation
		IFN-gamma	874 A/A (IFN-gamma)	Inflammation
Perez et al., 2019 [86]	383 ME/CFS	GPBAR1 (G protein-coupled bile acid receptor 1)	rs199986029	Macrophage functions and regulation of energy homeostasis by bile acids
		HLA-C (major histocompatibility complex, class I, C)	rs41560916	Immune system
		BCAM (basal cell adhesion molecule)	rs3810141	Intracellular signaling mediator
Carlo-Stella et al., 2009 [87]	75 CFS + 141 controls	RAGE (receptor for advanced glycation end-product)	Haplotypes: RAGE-374A, HLA-DRB1*1104 allele	Immunity and inflammation
		HLA-DRB1 (major histocompatibility complex, class II, DR beta 1)	RAGE-374A, HLA-DRB1*1301 allele	
Sommerfeldt et al., 2011 [88]	53 CFS (12–18 years)	COMT (catechol-O-methyltransferase)	AA genotype of SNP Rs4680 (COMT)	Catecholamine inactivation
		β₂-adrenergic receptor	CG and CC genotype of SNP Rs1042714 (β₂ -adrenergic receptor)	Catecholamine signaling

**Table 2:** Proposed biomarkers for CFS/MD differential diagnosis.

Ref.	Condition	Number of Participants	Markers Analyzed	MD	CFS	Potential Biomarker
Maes at al., 2012 [99]	Plasma pro-inflammatory cytokines	26 controls, 97 ME/CFS, 85 MD	IL-1	↓	↑	Plasma levels of IL-1 and TNF-α
			TNF-α	↓	↑	
Robertson et al., 2005 [100]	Lymphocyte subset	25 controls, 24 MD, 23 CFS	Resting T (CD3+/CD25-)	↓	↑	Resting T cells or CD20+/CD5+ B cells levels
			CD20+/CD5+ B cells	↑	↓	
Scott et al., 1999 [101]	Cortisol, adrenal androgens, DHEA, DHEA-S, 17-α-hydroxyprogesterone levels	11 controls, 15 MD, 15 CFS	DHEA	↑	↓	DHEA levels
Iacob et al., 2016 [102]	Exploratory factor analysis and regression analysis on 34 genes	61 controls, 31 medication-responsive MD 42 medication-resistant MD, 33 CFS	Purinergic and cellular modulators gene groups;	↓	↑	Purinergic, cellular modulators, nociception, and stress mediator gene expression analysis.
			Nociception and stress mediators group	↓	↑	
Morris et al., 2018 [103]	SPECT imaging	38 controls, 14 MD, 45 CFS	Mid cerebral uptake index	↑	↓	SPECT imaging abnormalities
Costa et al., 1995 [104]	SPECT imaging	40 controls, 29 MD, 67 CFS	Global and brainstem hypoperfusion	↓	↑	^99m^Tc-HMPAO SPECT differences
Goldstein et al., 1995 [105]	SPECT imaging	19 controls, 26 MD, 33 CFS	Dorsofrontal hypoperfusion	↓	↑	^99m^Tc-HMPAO SPECT imaging patterns of rCBF
			Right orbitofrontal lobe, left temporal lobe and left anterior frontal lobes hypoperfusion	↑	↓	
MacHale et al., 2000 [106]	SPECT imaging	15 controls, 12 MD, 30 CFS	Left prefrontal cortex hyperperfusion	↑	↓	^99m^Tc-HMPAO SPECT differences
			Left thalamus hyperperfusion	↓	↑	

MD: major depression; DHEA: dehydroepiandrosterone; DHEA-S: sulphate derivative of DHEA; SPECT: single-photon emission computerized tomography (also known as single-photon emission tomography (SPET)); CBF: cerebral blood flow; ^99m^Tc-HMPAO: (99m)Tc-hexamethyl-propylenamine-oxime. ↑: increased; ↓: decreased.

## Data Availability

Data sharing not applicable.
